# An analysis of time-varying dynamics in electrically sensitive arthropod hairs to understand real-world electrical sensing

**DOI:** 10.1098/rsif.2023.0177

**Published:** 2023-08-09

**Authors:** Ryan A. Palmer, Liam J. O’Reilly, Jacob Carpenter, Isaac V. Chenchiah, Daniel Robert

**Affiliations:** ^1^ School of Biological Sciences, University of Bristol, Life Sciences Building, 24 Tyndall Avenue, Bristol BS8 1TQ, UK; ^2^ School of Mathematics, University of Bristol, Fry Building, Woodland Road, Bristol BS8 1UG, UK

**Keywords:** mechanoreceptor, electroreception, electrostatic, sensory hairs, arthropods

## Abstract

With increasing evidence of electroreception in terrestrial arthropods, an understanding of receptor level processes is vital to appreciating the capabilities and limits of this sense. Here, we examine the spatio-temporal sensitivity of mechanoreceptive filiform hairs in detecting electrical fields. We first present empirical data, highlighting the time-varying characteristics of biological electrical signals. After which, we explore how electrically sensitive hairs may respond to such stimuli. The main findings are: (i) oscillatory signals (elicited by wingbeats) influence the spatial sensitivity of hairs, unveiling an inextricable spatio-temporal link; (ii) wingbeat direction modulates spatial sensitivity; (iii) electrical wingbeats can be approximated by sinusoidally modulated DC signals; and (iv) for a moving point charge, maximum sensitivity occurs at a faster timescale than a hair’s frequency-based tuning. Our results show that electro-mechanical sensory hairs may capture different spatio-temporal information, depending on an object’s movement and wingbeat and in comparison with aero-acoustic stimuli. Crucially, we suggest that electrostatic and aero-acoustic signals may provide distinguishable channels of information for arthropods. Given the pervasiveness of electric fields in nature, our results suggest further study to understand electrostatics in the ecology of arthropods and to reveal unknown ecological relationships and novel interactions between species.

## 1. Introduction

The study of an organism’s electrical interactions with its environment is a burgeoning field of research known as electric ecology. With a wealth of research in aquatic environments, e.g. [1,2], recent studies have moved into aerial and terrestrial environments. In the last decade, behavioural [3–6], electrophysiological [7] and environmental biophysical [8] studies demonstrated the importance of biological electrostatic interactions in arthropod ecology [9], and even atmospheric physics [8]. The above papers reveal how environmental electric fields could carry information about conspecifics, predators, prey, foraging resources, parasites, hosts, atmospheric conditions and perhaps more. For a broad survey of the literature on the ecology of electricity and electroreception, we refer readers to [9].

An understanding of receptor level processes is vital to appreciating the capabilities and limits of electroreception. To date, the only putative electrosensitive receptors in terrestrial arthropods are antennae (e.g. honeybees) and filiform hairs (e.g. bumblebees, hoverflies and spiders) [4–7]. Both receptors use mechanoreceptive systems that respond neurologically to movements driven by changes in local electric fields and the forces such variations generate. This motion occurs due to the receptor’s electrical properties, i.e. whether it possesses an electric charge and/or acquires charge by induction from local sources. An elegance of these receptors is their inherent multimodality (e.g. tactile and/or aero-acoustic stimuli), such that electrostatic stimuli can be transduced through the same sensory and neurological ‘hardware’, exploiting morphological substrates that may have co-evolved as multimodal sensors.

Regarding electrosensitive hairs specifically, recent work [7] on bumblebees provides evidence of electromechanical reception via sensory hairs and quantitative and model data for hair deflections and velocities under electrical stimuli. This work also shows neurological evidence of the action potentials generated in innervated hairs that are deflected by electrical stimuli. Since further empirical evidence at the receptor level is limited, theoretical studies are important for indicating future avenues of experimental exploration.

In this paper, we present such a theoretical examination of the response and sensitivity of electrically charged filiform hairs to electrical stimuli. While the quasi-static motion and spatial-sensitivity of such hairs have been previously studied [10–12], temporal sensitivity is considered here. The time-dependent response of sensory hairs to aero-acoustic cues has been extensively studied, mathematically and empirically, e.g. [13–20]. Within this aero-acoustic literature, filiform hairs are shown to tune to different frequencies (e.g. [13,17]). which determines the spatio-temporal sensitivity and motion of hairs. An outstanding question is whether electroreception and aero-acoustics have similar or distinct spatio-temporal sensitivities. This is a crucial question to answer in understanding the role of filiform hairs within an arthropod’s sensory ecology since the same sensory mechanism underpins the reception of both modalities (e.g. [7,11]). New spatio-temporal dynamics in terms of the hair motions are expected to arise in the context of electroreception. This expectation is partly due to the additional effect of electrostatic coupling (inter-hair forces from being charged). These forces may modify the hair motion further, differing from aerodynamic influences [11,12,21].

To this end, in §2, we discuss time-varying biological electrical signals. The spatio-temporal characteristics of such stimuli are considered and demonstrated by empirical data. In §3, we present a brief summary of the mechanics and modelling of electro-mechanical hairs, with details about the forces involved provided in §4. Our analysis begins in §5, where we assess the effect and role of a wingbeat in the sensory capacity of a hair. Subsequently, we consider the effect of a moving point charge in §6. To conclude our analysis, we examine the combined effects of a wingbeat and moving point charge in §7, comparing hair tuning and stimulus timescales. Finally, in §8, we comment on the possible biological implications of our results and avenues for future work.

## 2. Time-varying biological electrical signals

At a macro-scale, the net electric charge of an arthropod is predominantly static and unipolar. For example, a flying bumblebee charges triboelectrically through aerodynamic drag and friction or through collisions with ions. Bees typically become positively charged due to their position on the triboelectric series [22], tending to lose electrons when in contact with most other materials. At sufficient distance, a bee could be considered a point source of positive charge. Indeed, it may appear as such when measured rapidly through an induction ring (as in [23]) or a Faraday pail.

The electric field generated by an arthropod varies with time, due to its overall locomotion and the movement of charged body parts. For example, the beating of a flying bumblebee’s wings has a characteristic motion, within a certain frequency range determined by size, condition, flight trajectory, weight and speed. Such motion provides a spatio-temporal modulation of the electrical field, locally and globally.

**Figure 1.**
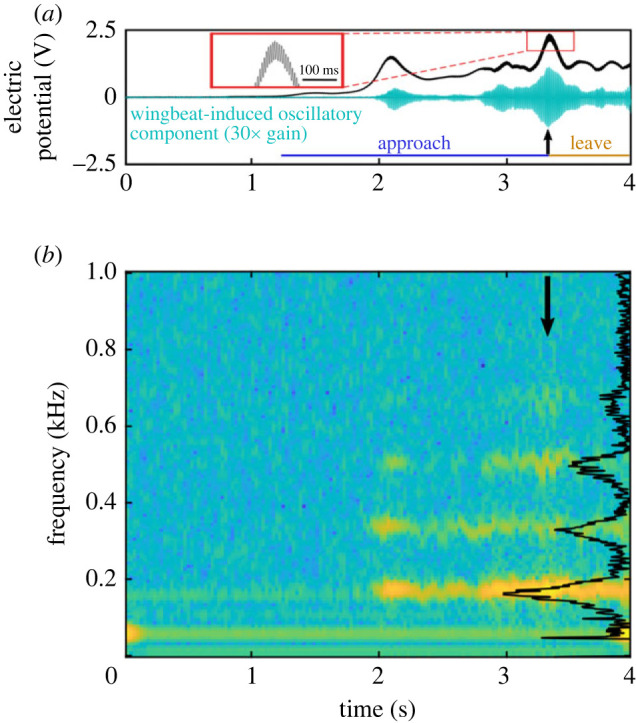
Non-contact measurement of a free flying buff-tailed bumblebee’s (*Bombus terrestris*) electrical potential as it approaches and leaves a fixed electrode. (*a*) Two traces show the spatio-temporal components of the bee’s electrical potential throughout its flight. The unfiltered signal (black) is dominated by (i) a quasi-static component that varies with the bee’s proximity to the electrode, and (ii) a smaller, yet significant, oscillatory component due to the bee’s wingbeat (its isolated waveform has been amplified 30×, shown by the teal curve). The approach and leaving phases are represented by blue and orange time lines, respectively, and the black arrow indicates the point of closest proximity to the electrode. (*b*) A spectrogram (Hamming window, 1024 lines) and power spectrum (Kaiser window) show the spectral content of the same recording. When the bee approaches the electrode, the dominant spectral content is its wingbeat frequency (165 Hz) and harmonics thereof.

The motion of charged wings contributes a detectable, dynamic component to the electrostatic signature of any flying insect. Greggers *et al.* [4] demonstrate this, showing that the honeybee waggle dance consists of ‘high’ and ‘low’ frequency electrical components. Complementary to this, we measured the local electrical potential of buff-tailed bumblebees (*Bombus terrestris*, Linnaeus 1758) free flying around a fixed electrode (figure 1).

The electrical signature’s direct current (DC)/extremely low-frequency components dominate the recordings, arising from the animal’s net charge and bulk movement. Additionally, the bee’s flapping wings contribute to the variation of the electrical field, adding an oscillatory component that grows in magnitude with proximity to the measuring electrode. This arises due to charge present on the wings and their varying distance. Importantly, it is expected that charge polarity does not change during this motion. Thus, wingbeat-driven, time-varying electrical signatures of flying insects are not alternating current (AC) signals and should be considered modulated DC (as first suggested in [4]) since their values never cross zero.

## 3. Morphology and mechanics of mechanoreceptor hairs

Arthropod filiform hairs exhibit broad morphological diversity between species and even within a single specimen [24,25]. Such differences arise, in part, from the hair’s function (e.g. mechanosensory, olfactory, thermal insulation). These morphological variations include branching and tapering; however, commonly, these hairs are comparatively long and thin. Such geometrical features inform how these hairs may be modelled.

Mechanosensory filiform hairs attach to arthropod bodies via viscoelastic socket membranes, enabling the hair to pivot in response to external forces [11,12,17,18,26,27]. Inside the basal socket is a mechanosensitive neurone that encodes suprathreshold hair deflections. Gathering such neural information from sometimes thousands of mechanosensory hairs, arthropods acquire and process valuable sensory information from their environment. Current evidence, e.g. [17,19,22,28], indicates that deflections required to elicit neural responses are typically small, *ca* 0.1°/10^−4^ rad, and that hair bending modes higher than the fundamental are not activated and therefore negligible. Hence, the hairs are considered to act as stiff rods.

Given the above morphological and mechanical features, we consider the hairs as a linear inverted pendulum (as is common in the literature, e.g. [11,17,18,29]). A hair’s motion is governed by

3.1 $$I_{h}{\ddot{\theta }}_{h}(t)+R_{h}{\dot{\theta }}_{h}(t)+S_{h}\theta_{h}(t)=-\tau_{h}(t),$$

where *τ*_*h*_(*t*) [kg m^2^ s^−2^] is the torque on the hair from external forces **F** [N m^−1^] acting perpendicular to the hair’s position **r**_*h*_ such that

3.2 $$\tau_{h}=|r_{h}\times F|.$$

Equation (3.1) describes a linear oscillator with moment of inertia *I*_*h*_ [kg m^2^] depending on the hair geometry (i.e. length and diameter) and density, while *R*_*h*_ [kg m^2^ s^−1^], the damping coefficient, and *S*_*h*_ [kg m^2^ s^−2^], the spring constant, relate to the viscoelastic socket membrane and vary depending on the arthropod [30,31].

The hair’s motion is defined within a given plane, taken to be the *x*–*y* plane, and hair deflections are measured from a resting position that aligns with the *y*-axis. For multiple hairs distributed in a line, the *h*^th^ hair tip location, *h* = 1, 2, …, *H*, is given by

3.3 $$\left(\begin{array}{c} x_{h}(t)\\ y_{h}(t)\\ z_{h}(t) \end{array}\right)=x_{h,0}+r_{h}(t)=\left(\begin{array}{c} x_{h,0}\\ y_{h,0}\\ 0 \end{array}\right)+\left(\begin{array}{c} L_{h}\sin(\theta_{h}(t))\\ L_{h}\cos(\theta_{h}(t))\\ 0 \end{array}\right),$$

where (*x*_0,*h*_, *y*_0,*h*_, 0) is the hair base. We assume that a hair carries a charge, *q*_*h*_, all located at the tip.

Hair motion occurs due to a force exerted on the hair tip, **F**. The precise details of this force depend on the source. In the proceeding sections, we restrict the force to the *x*–*y* plane so that the force and the hairs are co-planar. This restriction is reasonable since only force components perpendicular to the hair within the plane act to move the hair.

By treating the hairs as forced, damped harmonic oscillators (3.1), their motion can be described by their angular displacement, (*θ*_*h*_ [rad]), angular velocity, (${\dot{\theta }}_{h}\;[\text{rad}\;s^{-1}]$) and angular acceleration, (${\ddot{\theta }}_{h}\;[\text{rad}\;s^{-2}]$), about the pivot point [32].

## 4. Hair forces

In determining the hair motion, three forces linearly combine to produce **F** in (3.2): (i) aerodynamic forces, (ii) inter-hair electrical forces and (iii) external electrical forces.

### 4.1. Aerodynamic forces

Under a dynamic stimulus, the hair moves with non-zero angular velocity and acceleration. Therefore, aerodynamic forces act on each hair due to the air surrounding them opposing their motion.

To characterize the aerodynamic effects, consider the Reynolds number *Re* = *V*_*r*_ *d*_*h*_/*ν* of the fluid–hair interaction, where *V*_*r*_ = |*V*_*f*_ − *V*_*h*_| [m s^−1^] is the relative air velocity (*V*_*f*_) to the hair’s velocity ($V_{h}=y\dot{\theta }$, with *y* the distance from the substrate), *d*_*h*_ is the hair diameter (for hair lengths in the order of 1 [mm], typical diameters are 5 − 10 [μm]) and *ν* is the kinematic viscosity of air (*ν* = 1.562 × 10^−5^ [m^2^ s^−1^] at 25°C). In the proceeding analyses, *V*_*f*_ = 0 since the fluid does not provide a driving force (but does provide a resistive force). Hence, for *V*_*h*_ in the order of 10^−1^, *Re* = *O*(0.1) and viscous forces dominate.

Following [20], fluid drag and added mass forces from the surrounding fluid opposes the hair motion and induce a torque as follows:

4.1 $$\tau_{h}=-\int_{0}^{L}\left(\frac{\pi \mu }{2}y\dot{\theta }+\frac{\pi \rho d^{2}}{4}y\ddot{\theta }\right)y\;dy,=-\frac{\pi \mu L^{3}}{6}\dot{\theta }-\frac{\pi \rho d^{2}L^{3}}{12}\ddot{\theta }=-R_{\mu }\dot{\theta }-I_{\rho }\ddot{\theta }.$$

The fluid–hair interaction thus serves to effectively alter the hair’s moment of inertia (*I*) and damping (*R*) as functions of the fluid’s kinematic and dynamic viscosity. In effect, the hair’s tuning changes within a given medium (since tuning is governed by *I*, *R*, *S*). Therefore, a relative moment of inertia ${\tilde{I}}_{h}=I_{h}+I_{\rho }$ and a relative damping ${\tilde{R}}_{h}=R_{h}+R_{\mu }$ can be defined to incorporate the fluid’s influence in (3.1) as follows:

4.2 $$\begin{array}{rl} -\tau_{h}(t) & =(I_{h}+I_{\rho }){\ddot{\theta }}_{h}(t)+(R_{h}+R_{\mu }){\dot{\theta }}_{h}(t)+S_{h}\theta_{h}(t),\\ & ={\tilde{I}}_{h}\ddot{\theta }(t)+{\tilde{R}}_{h}\dot{\theta }(t)+{\tilde{S}}_{h}\theta (t). \end{array}$$

We denote ${\tilde{S}}_{h}=S_{h}$ for consistent notation.

### 4.2. Electrostatic forces

Consider *h* = 1, 2, …, *H* hairs in an array. Under no force, each hair remains at its resting position *θ*_*h*,0_. However, they may interact with neighbouring charged hairs, causing mutual deflections through Coulomb forces, denoted **F**_*h*,*i*_. Hence, the hair positions are initially located at equilibrium positions denoted **x**_*h*,*e*_ = (*x*_*h*,*e*_, *y*_*h*,*e*_, 0) as determined by an equilibrium angle, *θ*_*h*,*e*_.

If an external electrical field also acts on the hairs a force **F**_*h,E*_ is introduced. Hence, a hair’s response is determined by external and inter-hair forces that change as the hairs move. The general equation of motion for the *h*th hair in an array of *H* hairs is

4.3 $${\tilde{I}}_{h}{\ddot{\theta }}_{h}(t)+{\tilde{R}}_{h}{\dot{\theta }}_{h}(t)+{\tilde{S}}_{h}\theta_{h}(t)=-|r_{h}\times (F_{h,E}+F_{h,i})|,$$

consisting *H* simultaneous equations.

Now consider hair motions under two time-varying electrical fields: an oscillating point charge and a moving point charge. In the context of biological hair sensing, it is natural to consider both types of time-variation as discussed above and below. The general equation for the time-dependent force is

4.4 $$F_{h,E}(t)=k_{e}\frac{q_{p}q_{h}}{\|x_{h}(t)-x_{\;p}(t){\|}^{3}}\left(\begin{array}{c} x_{h}(t)-x_{\;p}(t)\\ y_{h}(t)-y_{\;p}(t)\\ 0 \end{array}\right),$$

where *k*_*e*_ ≈ 8.988 × 10^9^ [N m^2^ C^−2^] is the Coulomb constant and **x**_*h*_(*t*) given by (3.3).

#### 4.2.1. Oscillating point charge

Acoustic stimuli are oscillatory, possessing a frequency, magnitude and direction. The extensive literature on flow sensing via filiform hairs shows how these sensors tune to acoustic stimuli, with such as signals emanating from sources such arthropod noises and wingbeats. This latter example has an electrical analogue with the wingbeat of a charge-carrying arthropod introducing an oscillatory component into its electrical field (cf. figure 1). To model a wingbeat, we consider a point charge of fixed charge magnitude oscillating in space as follows:

4.5 $$x_{\;p}(t)=\left(\begin{array}{c} x_{\;p,0}+d\sin(\omega t)\cos(\phi )\\ y_{\;p,0}+d\sin(\omega t)\sin(\phi )\\ 0 \end{array}\right).$$

The wingbeat is described by a magnitude *d* [m], angular frequency *ω* and direction *ϕ*. Thus, a point charge of magnitude *q*_*p*_ [C] is considered to oscillate about the position (*x*_*p*,0_, *y*_*p*,0_, 0) [m].

#### 4.2.2. Moving point charge

To model a moving point charge, the frame of reference is centred on an observer about which an external stimulus moves. This is akin to an insect passing a leaf-dwelling arthropod. In this instance, the point charge moves as follows:

4.6 $$x_{\;p}(t)=\left(\begin{array}{c} x_{\;p,0}+f_{x}(t)\\ y_{\;p,0}+f_{y}(t)\\ 0 \end{array}\right),$$

where *f*_*x*_(*t*) and *f*_*y*_(*t*) describe the path of the point charge as functions of time. The position (*x*_*p*,0_, *y*_*p*,0_, 0) is the initial point charge location.

### 4.3. Equations of motion

The equations of hair motion under time-varying electrical fields are given by (3.3), (4.3) and (4.4) with either (4.5) or (4.6). In general, these are written as

4.7 $$\begin{array}{rl} & {\tilde{I}}_{h}{\ddot{\theta }}_{h}(t)+{\tilde{R}}_{h}{\dot{\theta }}_{h}(t)+{\tilde{S}}_{h}\theta_{h}(t)\\ & \;=k_{e}\;q_{h}L_{h}(\sum_{i\neq h}^{N}q_{i}\frac{\left(x_{h}(t)-x_{i}(t))\cos(\theta_{h}(t))-(y_{h}(t)-y_{i}(t))\sin(\theta_{h}(t)\right)}{({\left(x_{h}(t)-x_{i}(t)\right)}^{2}+{\left(y_{h}{\left(t)-y_{i}(t)\right)}^{2}\right)}^{3/2}}\\ & \;+q_{\;p}\frac{\left(x_{h}(t)-x_{\;p}(t))\cos(\theta_{h}(t))-(y_{h}(t)-y_{\;p}(t))\sin(\theta_{h}(t)\right)}{({\left(x_{h}(t)-x_{\;p}(t)\right)}^{2}+{{\left(y_{h}(t)-y_{\;p}(t)\right)}^{2})}^{3/2}}). \end{array}$$

We non-dimensionalize (4.7), to reduce the number of free parameters and simplify the analysis using the following scalings:

$$\begin{array}{rl} & \left(L_{h},{\tilde{I}}_{h},{\tilde{R}}_{h},{\tilde{S}}_{h},x_{h},q_{h},x_{\;p},q_{\;p},t\right)\\ & \;=(L{\hat{L}}_{h},I{\hat{I}}_{h},R{\hat{R}}_{h},S{\hat{S}}_{h},L{\hat{x}}_{h},q{\hat{q}}_{h},L{\hat{x}}_{\;p},q{\hat{q}}_{\;p},T_{c}\hat{t}). \end{array}$$

The following non-dimensional quantities are expected to be of order unity, unless otherwise stated.

While the above physical parameters can be readily scaled, the timescale depends on the nature of the source (e.g. wingbeat or moving point charge). Continuing with a general timescale, $t=T_{c}\hat{t}$, the *h*^th^ hair’s motion becomes

4.8 $$\begin{array}{rl} & {\ddot{\hat{\theta }}}_{h}(\hat{t})+A_{h}{\dot{\hat{\theta }}}_{h}(\hat{t})+B_{h}{\hat{\theta }}_{h}(\hat{t})\\ & \;=C_{h}(\sum_{i\neq h}^{N}{\hat{q}}_{i}\frac{\left({\hat{x}}_{h}(\hat{t})-{\hat{x}}_{i}(\hat{t}))\cos({\hat{\theta }}_{h}(\hat{t}))-({\hat{y}}_{h}(\hat{t})-{\hat{y}}_{i}(\hat{t}))\sin({\hat{\theta }}_{h}(\hat{t})\right)}{({\left({\hat{x}}_{h}(\hat{t})-{\hat{x}}_{i}(\hat{t})\right)}^{2}+{{\left({\hat{y}}_{h}(\hat{t})-{\hat{y}}_{i}(\hat{t})\right)}^{2})}^{3/2}}\\ & \;+{\hat{q}}_{\;p}\frac{\left({\hat{x}}_{h}(\hat{t})-{\hat{x}}_{\;p}(\hat{t}))\cos({\hat{\theta }}_{h}(\hat{t}))-({\hat{y}}_{h}(\hat{t})-{\hat{y}}_{\;p}(\hat{t}))\sin({\hat{\theta }}_{h}(\hat{t})\right)}{({\left({\hat{x}}_{h}(\hat{t})-{\hat{x}}_{\;p}(\hat{t})\right)}^{2}+{{\left({\hat{y}}_{h}(\hat{t})-{\hat{y}}_{\;p}(\hat{t})\right)}^{2})}^{3/2}}), \end{array}$$

with non-dimensional parameters

4.9 $$A_{h}=\frac{{\hat{R}}_{h}}{{\hat{I}}_{h}}\frac{R}{I}T_{c},\;B_{h}=\frac{{\hat{S}}_{h}}{{\hat{I}}_{h}}\frac{S}{I}T_{c}^{2}\;\mathrm{and}\;C_{h}=\frac{{\hat{q}}_{h}{\hat{L}}_{h}}{{\hat{I}}_{h}}\frac{k_{e}q^{2}}{IL}T_{c}^{2}.$$

## 5. An oscillating point charge

Our analysis begins by considering an oscillating point charge to understand the spatio-temporal effects of electrostatic wingbeats on sensory hairs. Following (4.8), the natural choice for the characteristic timescale is the period of oscillation for the stimulus *T* = 2*π*/*ω*. However, this is insufficient since a second timescale arises from the hair tuning.

Treating the hairs as linear oscillators, each has a natural frequency, $\omega_{h,0}=\sqrt{{\tilde{S}}_{h}/{\tilde{I}}_{h}}$, at which an unforced hair oscillates when disturbed from rest. The timescale of the oscillating driving force in (4.9) is therefore defined relative to the hair’s tuning

5.1 $$T_{c}=T_{\omega }=\frac{2\pi }{\omega }=\frac{2\pi }{r_{f}\omega_{0}},$$

where *r*_*f*_ = *ω*/*ω*_0_ and $\omega_{0}=\sqrt{S/I}$ is the scaled natural frequency. Substituting into (4.8) and rearranging, *A*_*h*_, *B*_*h*_ and *C*_*h*_ become

5.2 $$\begin{array}{r} A_{h}=\frac{4\pi }{r_{f}}\zeta_{h}{\hat{\omega }}_{h},\;B_{h}=\frac{4\pi^{2}}{r_{f}^{2}}{\hat{\omega }}_{h}^{2}\;\mathrm{and}\\ \;C_{h}=\frac{4\pi^{2}}{r_{f}^{2}}{\hat{\omega }}_{h}^{2}\frac{{\hat{q}}_{h}{\hat{L}}_{h}}{{\hat{S}}_{h}}K, \end{array}$$

where $\zeta_{h}={\tilde{R}}_{h}/2\sqrt{{\tilde{I}}_{h}{\tilde{S}}_{h}}$ is a hair’s damping ratio that characterizes dynamic behaviour, ${\hat{\omega }}_{h}=\sqrt{{\hat{S}}_{h}/{\hat{I}}_{h}}$ and *K* = *k*_*e*_ *q*^2^/*SL* is a non-dimensional parameter of electromechanical sensitivity for the system studied in [11]. Additionally

5.3 $${\hat{x}}_{\;p}(\hat{t})=\left(\begin{array}{c} {\hat{x}}_{\;p,0}+D\sin(2\pi \hat{t})\cos(\phi )\\ {\hat{y}}_{\;p,0}+D\sin(2\pi \hat{t})\sin(\phi )\\ 0 \end{array}\right),$$

for *d* = *DL*. By writing the system in this manner, the values of $r_{f},\zeta_{h},D,{\hat{\omega }}_{h},{\hat{q}}_{h},{\hat{L}}_{h},{\hat{S}}_{h}$, *K* and the spacing between hairs need to be set to analyse the system.

We restrict our attention to the roles of *r*_*f*_, *ζ*_*h*_ and *D*. These parameters have the largest effect on the spatio-temporal dynamics of sensory hairs. Notably, the effects of *K*, *L*_*h*_ and the hair spacing have been studied in [12]. The results and trends therein continue to hold in the current time-varying scenario presenting little additional effect. Furthermore, *q*_*h*_ and *S*_*h*_ act as scaling parameters and are set to one.

The damping ratio *ζ*_*h*_ characterizes the hair’s dynamic behaviour as follows:

— No damping (*ζ* = 0): the system never returns to rest. This is not observed in nature.
— Resonant under-damping $\zeta_{h}<1/\sqrt{2}$: increased deflection amplitudes occur when driven at or near a certain frequency ($\omega_{h,f}=\omega_{h,0}\sqrt{1-2\zeta_{h}^{2}}$) compared with the system’s response to frequencies far from the resonant value or at higher damping ratios.
— Non-resonant under-damping ($1/\sqrt{2}<\zeta <1$): a perturbed system oscillates as it returns to rest at a rate slower than a critically damped system. It does not show resonant behaviour when driven.
— Critical damping (*ζ* = 1): a perturbed system returns at the fastest rate, theoretically without oscillation.
— Over-damping (*ζ* > 1): a perturbed system returns to rest without oscillation at a slower rate than a critically damped system.

By specifying values *r*_*f*_ and *ζ*_*h*_, we assess how a hair’s spatio-temporal sensitivity to oscillating electrical signals varies for frequencies near to and far from its natural frequency and for different damping regimes.

### 5.1. Electrical sensing with a single hair

Sensitivity contours, developed in [11], are used throughout this section to assess the role of different parameters on a hair’s sensitivity and response. A sensitivity contour shows the locations (*x*_*p*_, *y*_*p*_) at which a point charge of magnitude *q*_*p*_ deflects a hair to a threshold *θ*_*s*_ [rad]. We set *θ*_*s*_ = 0.001 rad, which is representative of the minimum deflection required to elicit a neural response in several arthropods (crickets: 0.0032 rad [33], spiders: 0.0016 rad [19], bumblebees: 0.0007 rad [7]). Varying this threshold changes the contour size according to an inverse square law (when *θ*_*s*_ remains small), e.g. if *θ*_*s*_ is four times smaller, the contours double in all directions.

**Figure 2.**
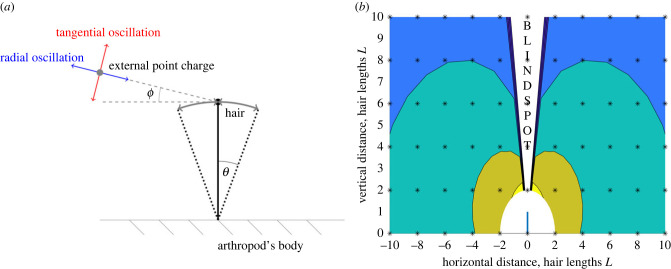
(*a*) Illustration of hair deflection due to an oscillating point charge. The point charge oscillates radially along or tangential to a line defined by an angle *ϕ* between the charge and hair tip position at rest. The hair oscillates in response, periodically producing a maximal deflection. (*b*) The response of a hair is modelled in response to single oscillating point charges placed at each location on a chosen grid (black asterisks). The resulting deflection data are plotted as a contour map showing the maximal deflection achieved by charges at each point. The curves show the locations of a point charge that deflect the hair to different thresholds (yellow-blue shows *θ*_*s*_ ≥ [1, 0.1, 0.01, 0.001]).

Figure 2 depicts the method for calculating sensitivity contours. Point charges along the contour curve, *θ*_*h*_ = *θ*_*s*_, and within the curve, *θ*_*h*_ > *θ*_*s*_, are considered detectable. Point charges outside the curve are considered undetectable, *θ*_*h*_ < *θ*_*s*_.

Two modes of wingbeat are now considered, denoted ‘radial’ or ‘tangential’, as illustrated in figure 2. To understand this terminology, consider a circle centred at the hair tip resting location with the arthropod on the circumference. Radial wingbeats oscillate in a direction along the circle’s radius while tangential wingbeats along the circle’s tangent. More formally, the direction of radial wingbeats is along a line connecting its mean position (*x*_*p*,0_, *y*_*p*,0_) and the hair tip at rest (*x*_*h*,0_, *y*_*h*,0_) = (0, 1). In (5.3), a radial wingbeat is defined for *ϕ* as

$$\phi =\arctan\left(\frac{y_{\;p,0}-y_{h,0}}{x_{\;p,0}-x_{h,0}}\right)=\arctan\left(\frac{y_{\;p,0}-1}{x_{\;p,0}}\right).$$

The direction of tangential wingbeats is perpendicular to this radial line, such that

$$\phi =\arctan\left(\frac{y_{\;p,0}-y_{h,0}}{x_{\;p,0}-x_{h,0}}\right)-\frac{\pi }{2}=\arctan\left(\frac{y_{\;p,0}-1}{x_{\;p,0}}\right)-\frac{\pi }{2}.$$

**Figure 3.**
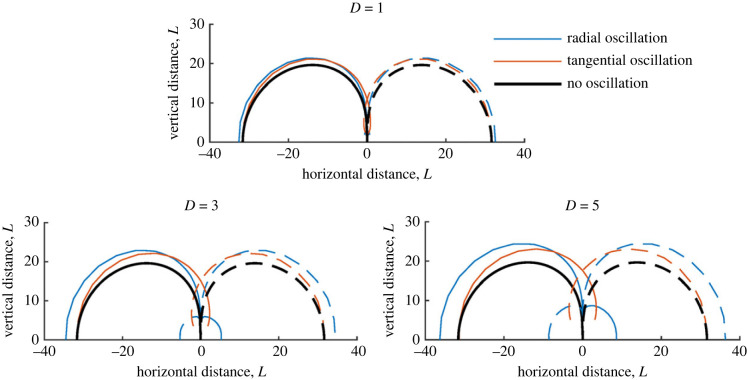
Sensitivity contours of a single hair with *ζ*_*h*_ = 0.5 for varying magnitude (*D*) and direction of wingbeat.

**Figure 4.**
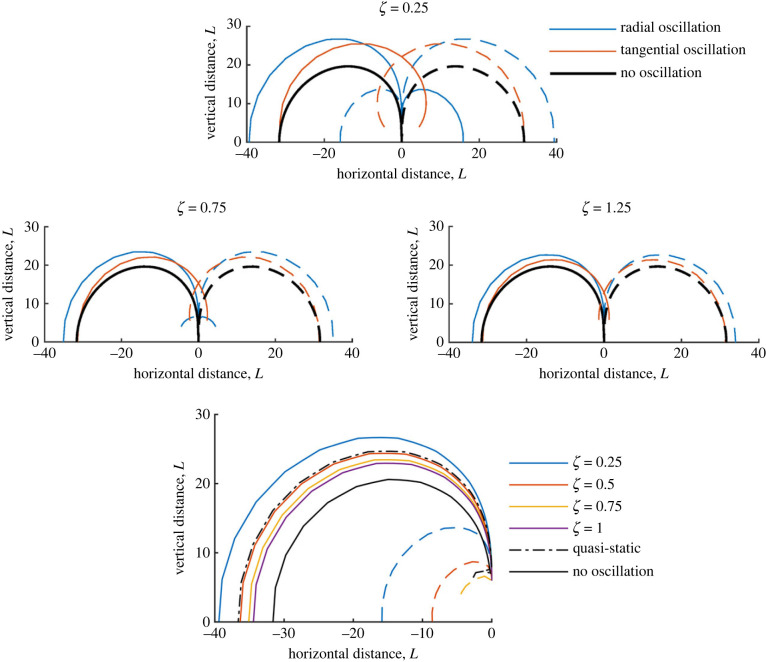
Sensitivity contours of a single hair for varying damping ratio (*ζ*_*h*_ = 0.25, 0.75, 1.25) and direction of wingbeat. The bottom sub-figure illustrates the variation in contour size with *ζ* = 0.25, 0.5, 0.75, 1 on the same axes, for a radial wingbeat. Also shown is the hair response for a quasi-static hair (*I*_*h*_ = *R*_*h*_ = 0) and the case without oscillation.

**Figure 5.**
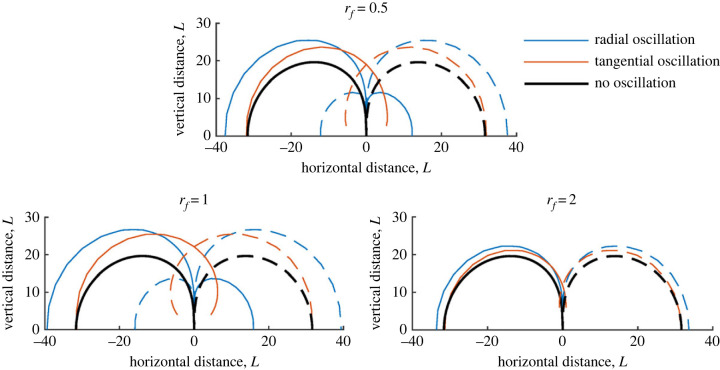
Sensitivity contours of a single hair with *ζ*_*h*_ = 0.25 for varying driving frequency (*r*_*f*_ = 0.5, 1, 2) and direction of wingbeat.

**Figure 6.**
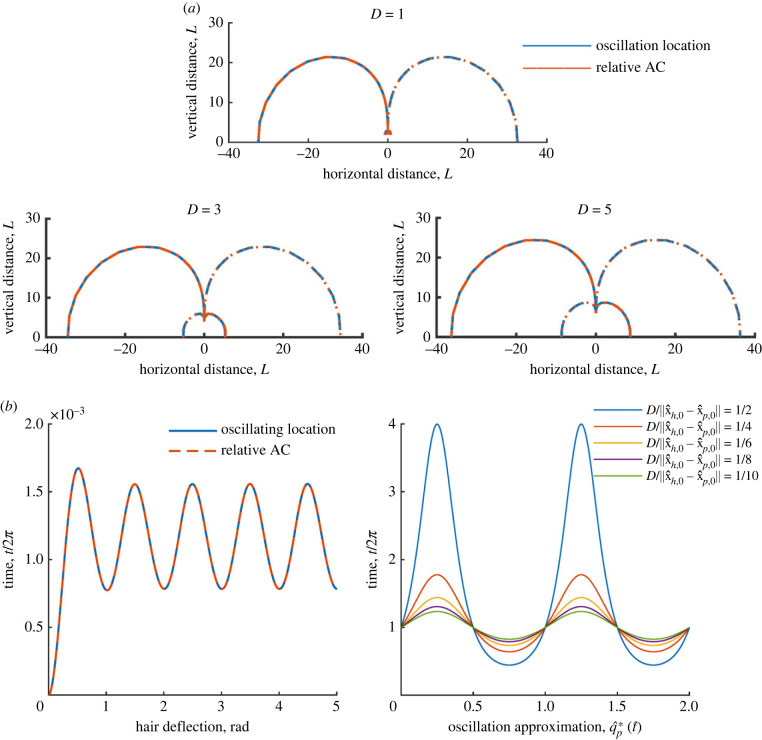
Comparison of how an oscillating point charge and an equivalent modulated DC signal affect a sensory hair. (*a*) Sensitivity contours of a single hair with *ζ*_*h*_ = 0.25 for a spatially oscillating radial wingbeat (blue) compared with a modulated DC approximation (fixed location and varying charge magnitude, red). (*b*) Left: example of time-varying hair motion for an oscillating point charge (blue) and for an equivalent modulated DC signal (red). Right: the modulated DC signal versus time for varying *D*.

Figures 3–6 show contours for a single hair, with different parameters varied in each. Radial and tangential oscillations are shown by blue and red curves, respectively. The black lines show the solution without a wingbeat for comparison. Unless otherwise stated, the wingbeat magnitude *D* = 5 while all other parameters equal one (*D* represents half a wingbeat and is non-dimensionalized by hair length and thus understood as a number of hair lengths).

#### 5.1.1. Varying wingbeat magnitude, *D* and direction

In figure 3, three plots show how the spatio-temporal sensitivity of a hair varies for *D* = 1, 3 and 5 hair lengths, respectively. For typical biological hairs, *L* = 1 [mm] and these values of *D* represent the wingbeat of several flying arthropods. Overall, the wingbeat’s direction and magnitude substantially change a hair’s response. The hair’s spatial sensitivity increases with *D* as shown by wider and higher contours, with radial and tangential wingbeats increasing the hair’s sensitive range compared with the non-oscillating case highlighting temporal sensitivity.

Comparing the different modes of oscillation, radial oscillations increase the hair’s horizontal sensitivity (by a distance greater than *D*) with tangential oscillations showing little effect here (e.g. when |*x*_*p*_| > 20, *y*_*p*_ ≤ 10). This is expected given the contour’s shape in this region. Closer to the hair in the *x*-direction, both modes have a similar magnitude effect on vertical sensitivity; however, tangential oscillations increase hair sensitivity in the ‘blindspot’ region (the region above the hair where the contours meet the hair tip and quickly diminish in sensitivity [11]). Most interestingly, and seen most strongly for *D* = 5, a new region of sensitivity occurs close to the hair, with significant positive and negative deflections. These regions are too close to the hair to be considered of sensory benefit for the given parameters; however, they highlight the overall change in hair dynamics and temporal sensitivity. Hence, the inclusion of wingbeats can increase the range of spatial sensitivity by a value greater than *D* due to the hair’s temporal sensitivity.

#### 5.1.2. Varying damping ratio, *ζ*_*h*_, and driving frequency

In figure 4, the effect of damping ratio on a hair’s spatio-temporal sensitivity to a wingbeat is shown for three oscillator types: resonantly under-damped (*ζ* = 0.25), under-damped (*ζ* = 0.75) and over-damped (*ζ* = 1.25). When the system is under-damped, and particularly resonantly under-damped, the hair’s temporal sensitivity increases significantly, which is shown here to enhance the hair’s spatial sensitivity in all directions (for *ζ* = 0.25 this increase is by a value generally larger than *D* = 5). For the over-damped system, a small increase in sensitivity is seen; however, this is likely due to the increased proximity of the point charge when it oscillates closer to the hair and not a result of temporal sensitivity. In most cases, a quasi-static hair (i.e. *I*_*h*_ = *R*_*h*_ = 0) produces larger contours; however, this case is not of biological relevance here (notably, it serves as a good approximation for ‘slowly’ varying stimuli). Throughout the paper, we now consider the case *ζ* = 0.25 (unless stated otherwise) since the temporal-spatial effects are strongest.

In figure 5, *r*_*f*_, the driving frequency relative to the system’s natural frequency, is varied. Three plots display the contours for *r*_*f*_ = 0.5, 1 and 2, respectively. When *r*_*f*_ = 1, the hair exhibits the largest deflections, as is expected, up to a 30% increase. For *r*_*f*_ = 0.5, a moderate increase in sensitivity is seen compared with *r*_*f*_ = 2, where only a small increase is seen. These results further reveal the link between spatial and temporal sensitivity. Depending on the hair tuning and driving frequency (e.g. temporal sensitivity), the spatial sensitivity can increase nonlinearly in terms of the range of detection (shown by the contour size and geometry). Equally, instances where the hair tuning and driving frequency are mismatched, only small increases in sensitivity occur. The latter case is likely due to the closer proximity of the charge, rather than an enhancement due to a hair’s temporal sensitivity.

#### 5.1.3. Approximating a wingbeat with a modulated DC signal

To assess the effect of a wingbeat charge on a sensory hair experimentally, one method would be to physically move an electrode in the presence of a sensory hair. However, this method introduces inaccuracies since it is difficult to oscillate accurately and consistently. Additionally, undesirable aerodynamic effects may be introduced. Thus, we derive an electrical stimulus that replicates the electrical field of wingbeat and can be delivered by a spatially static electrode.

To formulate this stimulus, first note that only the radial wingbeat can be captured since the electrical field lines act radially. Consider the force acting on the hair tip due to the point charge

5.4 $${\hat{F}}_{\;p}(t)=Kq^{2}{\hat{q}}_{h}{\hat{q}}_{p}\frac{1}{\|{\hat{x}}_{h}(t)-{\hat{x}}_{p}(t){\|}^{3}}\left(\begin{array}{c} {\hat{x}}_{h}(t)-{\hat{x}}_{\;p,0}-D\sin(2\pi \hat{t})\cos(\phi )\\ {\hat{y}}_{h}(t)-{\hat{y}}_{\;p,0}-D\sin(2\pi \hat{t})\sin(\phi )\\ 0 \end{array}\right).$$

From the definition of *ϕ* in the radial setting

5.5 $$\cos(\phi )=\frac{x_{h,0}-x_{\;p,0}}{\left\|{\hat{x}}_{h,0}-{\hat{x}}_{\;p,0}\right\|}\;\mathrm{and}\;\sin(\phi )=\frac{y_{h,0}-y_{\;p,0}}{\left\|{\hat{x}}_{h,0}-{\hat{x}}_{\;p,0}\right\|}.$$

For deflections of 0.001 rad, a hair remains close to its resting position and can be approximated by ${\hat{x}}_{h}(\hat{t})\approx {\hat{x}}_{h,0}$. Thus

$$\|{\hat{x}}_{h}(t)-{\hat{x}}_{p}(t)\|=\|{\hat{x}}_{h,0}-{\hat{x}}_{\;p,0}\|(1-D\sin(2\pi \hat{t})).$$

Inserting into (5.4) produces

5.6 $${\hat{F}}_{\;p}(t)=Kq^{2}{\hat{q}}_{h}{\hat{q}}_{p}\frac{1}{\|{\hat{x}}_{h}(t)-{\hat{x}}_{p}(t){\|}^{3}}\left(\begin{array}{c} {\hat{x}}_{h}(t)-{\hat{x}}_{\;p,0}\\ {\hat{y}}_{h}(t)-{\hat{y}}_{\;p,0}\\ 0 \end{array}\right){\left(1-\frac{D\sin(2\pi \hat{t})}{\left\|{\hat{x}}_{h,0}-{\hat{x}}_{\;p,0}\right\|}\right)}^{-2}.$$

Therefore, the wingbeat’s effect on a hair can be replicated by a modulated-DC electrical field with a time-varying charge

5.7 $${\hat{q}}_{p}^{*}(\hat{t})={\hat{q}}_{p}{\left(1-\frac{D\sin(2\pi \hat{t})}{\left\|{\hat{x}}_{h,0}-{\hat{x}}_{\;p,0}\right\|}\right)}^{-2}.$$

The profile of this signal (5.7) is displayed in figure 6 alongside its effect on the hair system. The figure shows close agreement between the results for an oscillating charge and its approximation.

## 6. A moving point charge

We now analyse the influence of a moving point charge on a two-hair system. Of interest is how the hair responses change for different velocities and hair parameters.

Using (4.8), the characteristic timescale, *T*_*c*_, in (4.9) requires defining for this new scenario. The natural choice is *T*_*c*_ = *T*_*V*_ = *L*/*V*, which is the timescale of the point charge motion given by dividing the characteristic hair length with a velocity scale. Substituting into (4.8) and rearranging, the three non-dimensional parameters *A*_*h*_, *B*_*h*_ and *C*_*h*_ become

6.1 $$\begin{array}{r} A_{h}=\frac{2L}{V}\zeta_{h}\omega_{0}{\hat{\omega }}_{h,0},\;B_{h}=\frac{L^{2}}{V^{2}}\omega_{0}^{2}{\hat{\omega }}_{h,0}^{2}\;\mathrm{and}\\ \;C_{h}=\frac{L^{2}}{V^{2}}\omega_{0}^{2}{\hat{\omega }}_{h,0}^{2}\frac{{\hat{q}}_{h}{\hat{L}}_{h}}{{\hat{S}}_{h}}K, \end{array}$$

with $K,\omega_{0},{\hat{\omega }}_{h,0},\zeta_{h}$ defined as before. Additionally

6.2 $${\hat{x}}_{\;p}(\hat{t})=\left(\begin{array}{c} {\hat{x}}_{\;p,0}+{\hat{\;f}}_{x}(\hat{t})\\ {\hat{y}}_{\;p,0}+{\hat{\;f}}_{y}(\hat{t})\\ 0 \end{array}\right),$$

with ${\hat{\;f}}_{x}(\hat{t})$ and ${\hat{\;f}}_{y}(\hat{t})$ being trajectory functions. The previous system (5.2) can be compared with the current system (6.1) by defining a parameter *r* that compares the two timescales, e.g. (4.8) can be written with the following parameters:

6.3 $$\begin{array}{rl} & r=\frac{2\pi }{\omega_{0}}\frac{V}{L}\\ \mathrm{and}\; & A_{h}=\frac{4\pi }{r}\zeta_{h}{\hat{\omega }}_{h},\;B_{h}=\frac{4\pi^{2}}{r^{2}}{\hat{\omega }}_{h}^{2},\;C_{h}=\frac{4\pi^{2}}{r^{2}}{\hat{\omega }}_{h}^{2}\frac{{\hat{q}}_{h}{\hat{L}}_{h}}{{\hat{S}}_{h}}K, \end{array}\}$$

to incorporate the new timescale with *C*_*h*_ showing how the Coulomb force varies with 1/*V*^2^.

In the following analysis, we consider a point charge moving horizontally past the hair system such that: ${\hat{\;f}}_{x}(\hat{t})=\hat{t}$ and ${\hat{\;f}}_{y}(\hat{t})=0$. The point charge starts far from the array in the *x*-direction and 10 hair lengths vertically above the array, giving $\left({\hat{x}}_{\;p,0},{\hat{y}}_{\;p,0})=(-400,11\right)$. Therefore, its motion is defined as

6.4 $${\hat{x}}_{\;p}(\hat{t})=\left(\begin{array}{c} -400+\hat{t}\\ 11\\ 0 \end{array}\right).$$

**Figure 7.**
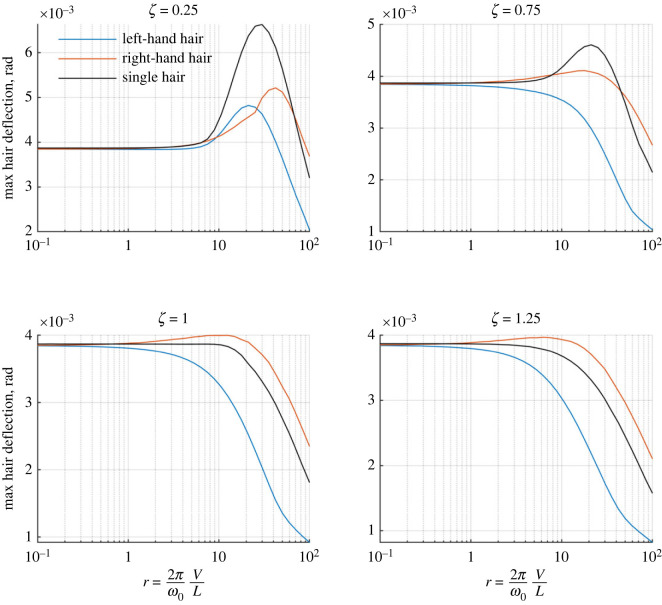
The maximum deflection of two coupled hairs in the presence of a moving point charge for varying *r* (6.3). Blue curves: left-hand hair, red curves: right-hand hair, black curves: single hair for comparison. Four oscillator regimes are presented and the hairs possess the same tuning with *ζ*_1_ = *ζ*_2_ = 0.25, 0.75, 1, 1.25.

In figure 7, we assess how the spatio-temporal sensitivity changes with *r* (e.g. varying the point charge’s velocity relative to the hair tuning). We compare two hairs with a single hair, using the maximum hair deflection in response to the moving charge as a measure of sensitivity.

Different hair responses and sensitivities are seen for each damping ratio considered. Overall, for *ζ* = 0.25 (resonantly under-damped), the largest amplitudes of deflection are seen for both the single and two-hair systems. The single hair undergoes significantly larger deflections for most values of *r*, indicating an inhibiting effect from the hair-hair coupling on the overall hair response. Though slightly diminished, the same trend is seen when *ζ* = 0.75 (non-resonantly under-damped). Increasing *ζ* further to critically damping (*ζ* = 1) and over-damping (*ζ* = 1.25), the two-hair system shows larger deflections in the right-hand hair compared with the single hair. The hair coupling is now enhancing the hair motion with the left-hand hair amplifying the second hair.

Most interestingly, peaks in the hair deflections for both systems occur when *r* = *O*(10). For an oscillating signal, under-damped hairs are most sensitive when the driving frequencies (and timescale) tends towards the system’s natural frequency system, e.g. *r*_*f*_ = 1. For over-damped hairs, hairs are most sensitive to driving frequencies that are much smaller than the natural frequency (e.g. quasi-static signals, *r*_*f*_ < 1). For a moving point charge, the hair’s sensitivity increases when the timescale of the charge’s movement is 10 times larger than that of its natural frequency, independent of the damping ratio. These results point to a potential difference in the utility of sensory hairs in detecting oscillating and moving electrostatic signals. They also show how the hair coupling may transition from an inhibiting to enhancing effect as the point charge moves faster.

**Figure 8.**
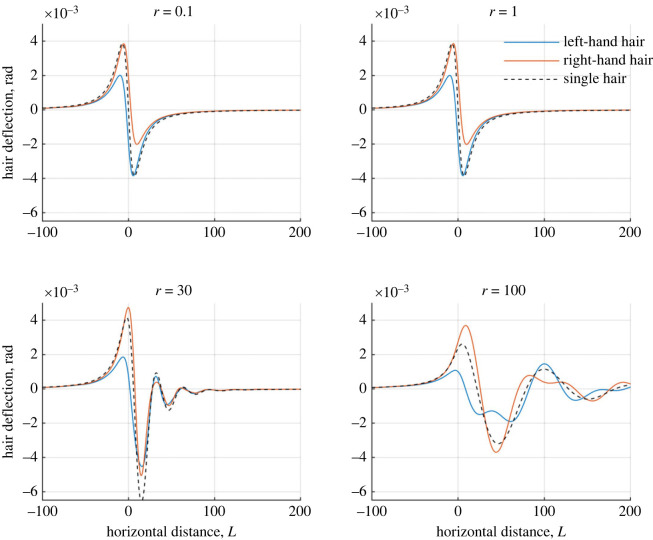
Plots showing how two hairs deflect in the presence of a moving point charge against the charge’s distance from the array centre for varying *r* (6.3). Blue curves: left-hand hair, red curves: right-hand hair, black curves: single hair for comparison. The hairs possess the same tuning with *ζ*_1_ = *ζ*_2_ = 0.25.

Examining these results further, figure 8 shows the hair responses as a point charge moves towards, over and past the array for *r* = 0.1, 1, 30 and 100 (at *r* = 30 the largest single hair deflections occur for *ζ*_*h*_ = 0.25). The results for *r* = 0.1 and *r* = 1 are very similar, with the two-hair system achieving a similar magnitude of deflections as the single hair system. Notably, the single hair covers the total two-hair response (e.g. the same maximum deflection as the right-hand hair and the same minimum deflection as the left-hand hair). When *r* = 30, the single hair has a greater negative deflection compared with the two-hair system, after the point charge has moved past the hairs. Small oscillations now occur as the hairs return to rest, with both hairs in phase with each other. Finally, when *r* = 100 the hair responses change distinctly. The single hair presents longer oscillatory behaviour as it returns to rest. For the two hairs, the inter-hair coupling comparatively enhances the right-hand hair’s deflection and generates irregular oscillations in both hairs, moving them out of phase with one another.

By way of explanation, when *r* = 0.1 and 1, the point charge has the smallest relative velocities. Therefore, the point charge remains in the vicinity of the hairs for longer, producing a stronger electrostatic force between the hairs and point charge (due to the proximity). This force dominates the interaction with the hair responses resembling a quasi-static motion.

The oscillations seen in the cases for *r* ≥ 30 are suppressed when *r* is small as the point charge’s force continues to dominate the hair motion after it has passed by, effectively damping the system. When *r* increases, the point charge moves past and away from the hairs more quickly, diminishing the force it exerts on the array faster. In this instance, the hairs can oscillate freely as they return to rest, which is the expected behaviour of a perturbed oscillator.

Finally, for *r* = 100, the dynamics are slightly more complex. Similar to *r* = 30, the single hair is perturbed and oscillates to rest. One period of oscillation occurs as the point charge moves from *x* = 0 to *x* = 100 as indicated by *r* = 100 (e.g. the point charge moves 100 hair lengths in the period of a single oscillation at the natural frequency). For the two-hair system, there is a greater disparity between the deflections of both hairs when the point charge passes the left-hand hair. This results in the hair tips being slightly further apart, affecting their inter-hair coupling as they now move out of phase and interfere with each other’s return to rest. (Similar results are seen for larger values of *ζ*, but with diminishing oscillations for bigger *r* values.)

## 7. A moving point charge with a wingbeat

Bringing the paper’s main threads together, we now consider a moving point charge with a wingbeat. There are three timescales to consider, those of the point charge’s movement, a hair’s natural frequency and the wingbeat frequency. Taking the characteristic timescale as the point charge’s movement relative to the system’s natural frequency, the non-dimensional parameters of (4.8) are again given by (5.2). The point charge’s motion is defined by

7.1 $${\hat{x}}_{\;p}(\hat{t})=\left(\begin{array}{c} {\hat{x}}_{\;p,0}+{\hat{\;f}}_{x}(\hat{t})+D\sin(2\pi \hat{t})\cos(\phi )\\ {\hat{y}}_{\;p,0}+{\hat{\;f}}_{y}(\hat{t})+D\sin(2\pi \hat{t})\sin(\phi )\\ 0 \end{array}\right)=\left(\begin{array}{c} {\hat{x}}_{\;p,0}+\frac{r}{r_{f}}\hat{t}+D\sin(2\pi \hat{t})\cos(\phi )\\ {\hat{y}}_{\;p,0}+D\sin(2\pi \hat{t})\sin(\phi )\\ 0 \end{array}\right),$$

where

$$\frac{r}{r_{f}}=\frac{2\pi }{r_{f}\omega_{0}}\frac{V}{L}$$

and

$$r=\frac{2\pi }{\omega_{0}}\frac{V}{L}\;\mathrm{and}\;r_{f}=\frac{\omega }{\omega_{0}}$$

as in (6.3) and (5.1), respectively.

**Figure 9.**
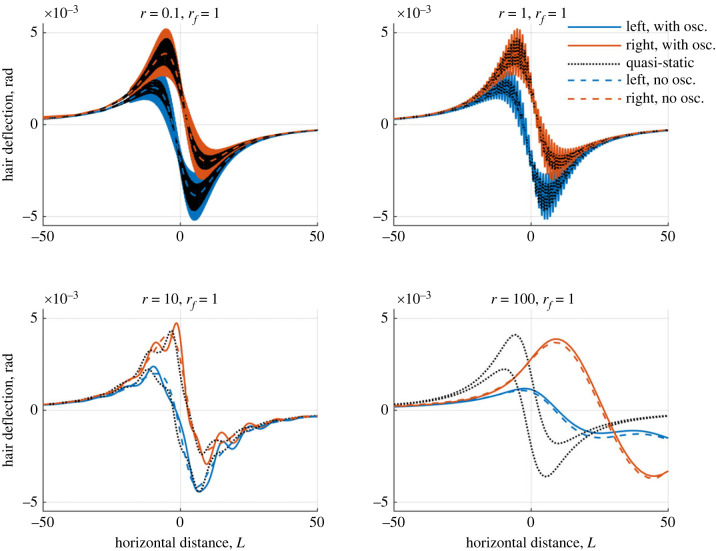
Plots showing how two hairs deflect in the presence of a moving point charge with wingbeats in the *y*-direction, for varying *r* (6.3). The horizontal axis is the charge’s distance from the array centre. Blue curves: left-hand hair, red curves: right-hand hair, black curves: single hair for comparison. Dashed lines show the responses without a wingbeat. The hairs possess the same tuning with *ζ*_1_ = *ζ*_2_ = 0.25.

**Figure 10.**
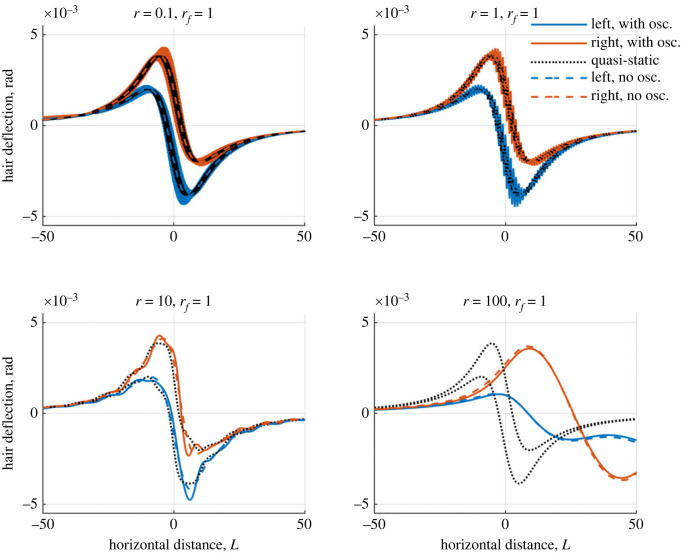
Plots showing how two hairs deflect in the presence of a moving point charge with wingbeats in the *x*-direction, for varying *r* (6.3). The horizontal axis is the charge’s distance from the array centre. Blue curves: left-hand hair, red curves: right-hand hair, black curves: single hair for comparison. Dashed lines show the responses without a wingbeat. The hairs possess the same tuning with *ζ*_1_ = *ζ*_2_ = 0.25.

In figures 9 and 10, the same scenario in figure 8 is analysed with wingbeats in the *y*-direction (vertical wingbeats with fixed *ϕ* = *π*/2 in (7.1)) and *x*-direction (horizontal wingbeats with fixed *ϕ* = 0 in (7.1)) relative to the point charge’s motion included, respectively. The point charge begins far from the hair array at ( −400, 11) and moves with scaled velocity *r* across the domain towards and past the hair array.

Four different flight speeds are modelled, *r* = 0.1, 1, 10 and 100. We consider *r* = 1, such that the wingbeat matches the hair’s natural tuning and the wingbeat’s magnitude is *D* = 1. The hairs have a damping ratio *ζ*_*h*_ = 0.25, *h* = 1, 2. The aim is to understand how the hair response changes with the inclusion of a wingbeat for different flight regimes and ascertain how different information is elicited in various scenarios.

For *r* = 0.1 and *r* = 1, the point charge moves 0.1 and 1 hair lengths per wingbeat respectively. There are two distinct components to the hair responses. Firstly, the point charge’s movement deflects the hairs similar to the non-oscillating charge. Hence, the underlying shape resembles that of figure 8. Secondly, the wingbeat causes further deflections, oscillating the hair about the underlying bulk deflection. Compared with the quasi-static case, the time-dependence serves to increase the amplitude of hair oscillations in response to the wingbeat. Larger oscillations are seen for the vertical wingbeat orientation (figure 9), while horizontal wingbeats (figure 10) shift the deflection peak. Overall, the two dynamic components show little interaction in terms of the hair response, remaining distinct.

When *r* increases to 10, the relative influence of the point charge’s velocity increases and the wingbeat becomes embedded in the deflection profiles in figures 9 and 10. The wingbeat is no longer distinct from the bulk deflection as seen for smaller *r*, revealing a stronger interaction between the dynamic components. In figure 9, the wingbeat shifts and increases the deflection magnitude slightly in comparison with the quasi-static and no oscillation cases. When *r* increases further the wingbeat’s influence decreases such that at *r* = 100 the profiles with and without oscillations roughly match.

**Figure 11.**
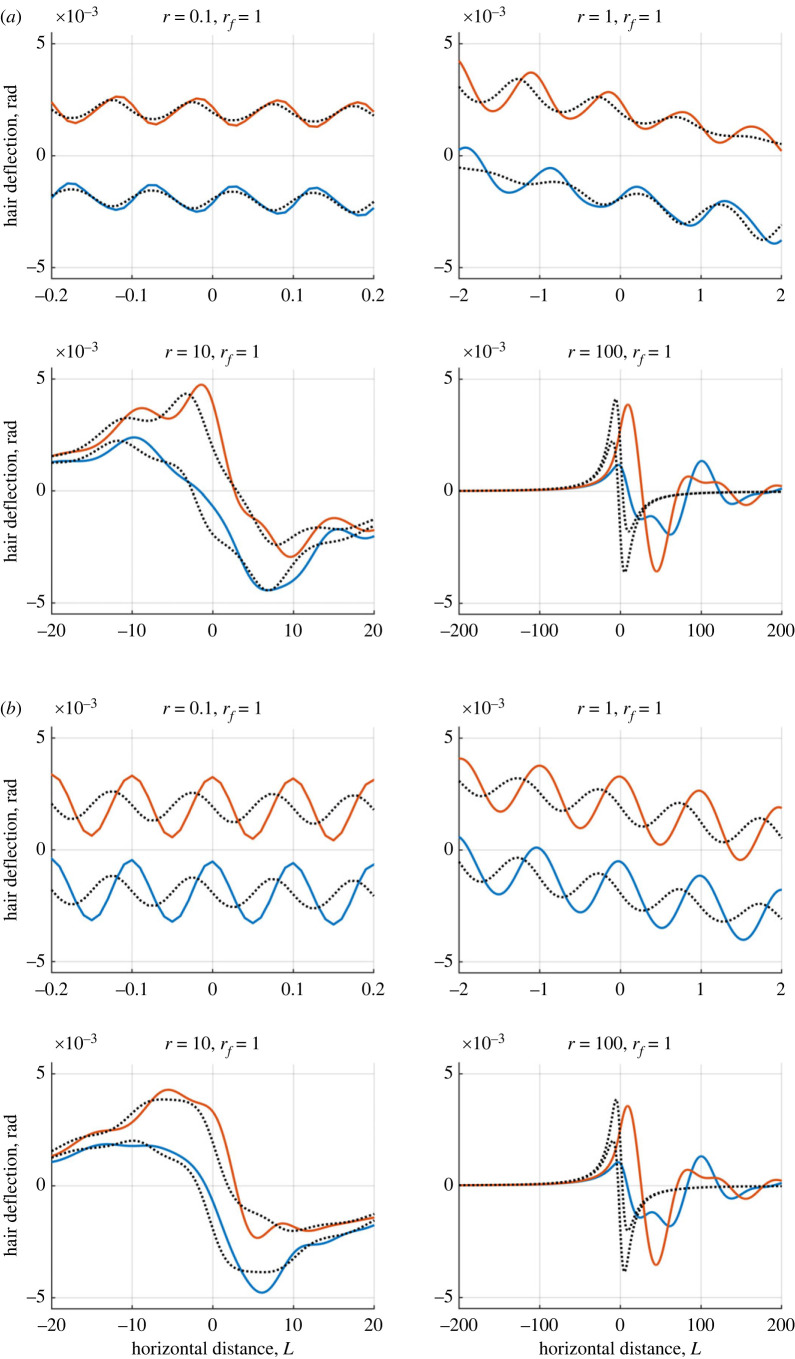
Curves showing the hair response and distance travelled by a point charge in a fixed time interval of four wingbeats with *D* = 1 about the array centre ($\hat{t}\in [-2,2]$). Blue curves: left-hand hair, red curves: right-hand hair, black curves: single hair for comparison. Dashed lines show the responses without a wingbeat. The hairs possess the same tuning with *ζ*_1_ = *ζ*_2_ = 0.25. (*a*) A close-up figure 9. (*b*) A close-up figure 10.

Figure 11 highlights the spatio-temporal scales by comparing the hair deflections and the point charge location over four wingbeats (centred about the hairs). In the same time span, the spatial (horizontal) scales of the point charge-hair interaction vary across each case, and the dominant dynamics change. These figures highlight different dynamic contributions. For small *r*, the wingbeat is the only discernible information, over a small horizontal distance. With increased *r*, larger amplitude dynamics associated with point charge movement dominate changing the hair response and thus the sensory information received. Notably, for *r* = 100 the hair deflection profiles in (*a*) and (*b*) are the same, further highlighting the diminished wingbeat influence.

## 8. Discussion

The time-dependent motion, interaction and sensitivity of charged mechanosensory hairs has been investigated. By analysing oscillatory wingbeats and moving point charges, we reveal new sensory dynamics and timescales that underlie electroreception.

The main findings are:

(1) Wingbeat frequency influences the spatial sensitivity of hairs, depending on the hair tuning as shown by sensitivity contours [11]. This modelling framework indicates how the time-dependent dynamics of the oscillator’s angular deflection, velocity and acceleration combine to alter the geometry of the contours.
(2) Wingbeat direction and magnitude alter the range and shape of spatial sensitivity. When oscillating radially, spatial sensitivity increases horizontally and vertically, while tangential wingbeats only effect vertical sensitivity.
(3) A radial wingbeat can be approximated using a sinusoidally modulated DC signal given by (5.7). This result allows for the effect of electrostatic wingbeats to be easily produced in empirical studies. Moving an electrode to mimic a wingbeat would introduce undesirable aerodynamic influences, and is hard to perform precisely. Using a fixed electrode to deliver the modulated-DC signal eliminates these issues.
(4) For a moving point charge, maximum sensitivity occurs at a faster timescale than the hair tuning. Across several cases, the hair deflection maximizes when the point charge’s motion is an order of magnitude faster than the hair tuning. Figures 9 and 10 indicate an important sensory distinction, in where, depending on the timescale, different components of the signal dominate, leading to the possible decoupling and discernment of signals through dynamic hair motions. Therefore, electro-mechanical sensory hairs may capture different spatio-temporal information depending on the velocity of the moving object and wingbeat frequency.

### 8.1. Biological implications

The analysis and results are non-dimensionalized ensuring they hold across scales. Whilst this representation is useful for a general analysis, it can be difficult to interpret the biological implications. To this end, we make several comments.

#### 8.1.1. The relevance of a time-varying electrical field

The time-varying components of a flying insect’s electrical signature could provide information useful to itself, conspecifics and heterospecifics alike. Quasi-static or low-frequency elements attributable to the net charge of a flying insect may provide information on its arrival and departure or presence and absence. The longer time period of such information could be used by prey species attempting to detect an approaching airborne predator. Coupled with higher frequency oscillations in the electric field from a predator’s wingbeat, more detailed information on the identity of the approaching predatory threat may be available, for instance, assessing whether it is a wasp or harmless passer-by.

Time-varying electrical components contribute to intraspecific communication through the honeybee waggle dance [4]. This is an example of direct electrical communication. Additionally, predators and parasites use acoustic cues to locate prey/hosts, e.g. [34,35], and if wingbeat frequencies are characteristic of certain taxa, electroreceptive parasites and predators could equally use these as electric location cues [21].

An electroreceptive flying insect may also use spectral information from its own wingbeat in its electroreceptive process. For example, when a charged bee approaches a flower, a charge of opposite polarity is induced in the flower to counter the electric field, an example of Faraday induction. When the induced charge is detectable, there may be two different timescales over which information can be gleaned. Firstly, the quasi-static changes induced by bulk movements over a longer distance may inform the bee of the flower’s presence. Secondly, when approaching the flower, oscillations in the polarized charge attributable to the wingbeat may become apparent. The speed at which these rapid changes induce opposite charge relate to a flower’s conductivity, with greater conductivity resulting in more rapid charging. In fact, different parts of a flower with different electrical properties will respond to the wingbeat with information-rich variation. Indeed, a flower lacking nectar may be less conductive and respond slower, providing a non-contact measure of the flower’s foraging value. Here, higher frequency time-varying components may offer subtle information on a shorter spatial scale.

#### 8.1.2. Time-varying sensitivity contours

Section 5 explores the spatio-temporal sensitivity of sensory hairs, showing the dependence of the hair response on signal frequency in figures 4 and 5 for single hairs. Variation in a hair’s sensitive range is clearly seen for different frequency stimuli (e.g. values of *r*_*f*_), indicating the possibility of enhanced proximity detection for certain frequencies and tunings. That is, wingbeat frequencies close to the hair tuning are detectable at greater distances than other frequencies (particularly higher frequencies). While under-damped systems show enhanced hair motion, wingbeats also modify responses in over-damped systems, showing benefit across hair systems.

When considered in time, the hair oscillates about a perturbed position. Thus, depending on how deflection information is neurologically encoded, a hair’s angular velocity and acceleration may provide further information and bestow further sensory utility when hair phases are considered.

#### 8.1.3. Moving point charges

For moving point charges with a wingbeat, we introduced the parameter

$$r=\frac{2\pi }{\omega_{0}}\frac{V}{L},$$

which compares the timescales of hair tuning and the point charge motion. Here, *r* can be interpreted as how many hair lengths the point charge moves in a single wingbeat.

To understand how these results inform the movement ecology of arthropods, consider the hair array to represent an arthropod dwelling on a substrate that observes a passer-by. The question is what information is captured by electro-mechanically sensitive hairs? Important sensory quantities include a passing arthropod’s location, velocity and wingbeat frequency. In predator–prey interactions, this key information is ideally discerned as quickly and as accurately as possible. Any delay can make a significant difference to successful predator avoidance or prey capture.

Interpreting the results in their biological context, the range of flights speeds (*r* = 0.1–100) presented in §§6 and 7 can be shown to cover a range of empirically verified speeds, as collated and presented in [36].

Given a hair length of 1 mm (relevant for many terrestrial arthropods [37]), and *ω*_0_ = 100 Hz (since arthropod filiform hairs often acoustically tune to frequencies in the order of 100 Hz, e.g. [17,20,29], coinciding with common wingbeat frequencies [14,29,38]), a value of *r* = 0.1 represents a point charge moving with velocity *V* = 0.01 m s^−1^ (e.g. *V* = *r* *L*/*T*_0_), which is in the realms of hover flight such as when a bee approaches a flower [39]. Hence, the results for *r* = 0.1 in figures 7–11 may be interpreted as an observer detecting an arthropod passing by in hover flight.

Several sensory dynamics are seen: (i) the wingbeat dominates the small-time dynamics (figures 9–11), (ii) there is significant sensitivity to the moving point charge (figures 7 and 8) with sensitivity spanning a longer timescale than the wingbeat (figure 11) and (iii) for the two-hair system, the hairs remain in phase with one another (figure 8).

When *r* = 1, the hair tuning and point charge velocity timescales are comparable with *V* = 0.1 m s^−1^. The dynamics (i), (ii) and (iii) above occur here, yet an important distinction is that the sensory information is conveyed more quickly. Notably, there is a wider horizontal acuity within the same time-period than *r* = 0.01.

For *r* = 10, the influence of the signal components coincide in the hair response. This scenario represents *V* = 1 m s^−1^ which is the flight speed for many arthropods (e.g. bumblebees, honey bees, calyptrate flies and hawk-moths). The oscillations of the wingbeat embed within the bulk deflection caused by point charge’s movement. Hence, it is harder to discern the wingbeat frequency. The hairs are now slightly out of phase with one another, introducing a new sensory dynamic with asymmetrical deflections, angular velocities and angular accelerations.

The final case, *r* = 100 relates to a flight speed of *V* = 10 m s^−1^, the fastest arthropod flight speeds (e.g. horseflies, dragonflies and locusts). The large discrepancy in the dynamic timescales makes the wingbeat mechanically undetectable. Additional hair oscillations occur when the point charge has long moved past the hair array (approx. 50–100 hair lengths in figure 11); and are similar to the case when *r* = 100 in figure 8 with no wingbeat considered. These oscillations occur when a perturbed oscillator returns to rest. Overall, the wingbeat dynamics play a potentially indistinguishable role here, indicating the limit of wingbeat detection from electrical stimuli.

The distinction in hair dynamics for each flight regime enables the acquisition of different sensory information. For *V* = 0.01–0.1 m s^−1^ (*r* = 0.1 − 1), the timescales for the wingbeat and point charge movement are most distinct. While the point charge’s horizontal motion causes a larger bulk deflection over a slower timescale, rapid yet smaller oscillations in the hair motion arise due to the wingbeat. These differences may enable an observer to disambiguate signals, and decouple different sensory components. The arthropod receiver may therefore isolate the point charge location (as suggested in [21]) over the slower timescale, and the wingbeat frequency based on the magnitude and angular velocities of a single hair, or indeed multiple hairs. For example, arrays of differently tuned hairs would further enable this frequency discrimination, as is seen in aero-acoustic sensing [38,40].

At faster velocities, this disambiguation becomes difficult. When the point charge moves faster than the wingbeat, the time frame in which the observer can process such information reduces and the wingbeat is no longer mechanically discernible. Yet, using the example of predator–prey interactions, an arthropod passing by at this speed and proximity is unlikely to be an immediate threat. Other more representative flight paths and speeds should be considered to assess this.

Filiform hairs vary in shape, size and tuning, hence we examined damping ratios from resonantly under-damped hairs to over-damped hairs to cover biologically likely sensors. Having mostly commented on resonant hairs, we now briefly discuss over-damped hairs. The response of over-damped hairs is significantly muted to oscillatory stimuli, often showing little difference to non-oscillatory cases (figure 3). However, in the detection of moving stimuli, two over-damped hairs show greater sensitivity compared with single hairs (figure 6). This indicates the possibility that over-damped hairs better detect bulk electrical stimuli than aero-acoustic/oscillatory signals. While further evidence is required, this adds to the conclusions raised in [11,41] around the potential for electro-specific or increased electro-sensitivity to emerge in hair systems through differences in morphology (e.g. length, stiffness), enabling multi-modal sensing via many hairs.

As further comment on the bimodality of sensory hairs, we suggest that sensing both electrostatic and aero-acoustic stimuli may greatly enhance an arthropod’s proximity detection, localization and frequency discrimination. This is due to the strength, direction and influence of each modality differing (depending on the scenario) due to their distinct underlying physics, and the possibility that aero-acoustic and electrostatic hair sensing could be optimized for different sensory ecological contexts and spatio-temporal scales. While further empirical evidence is required, the possibility exists that each modality provides a unique and distinguishable channel of information. The pervasive presence of this electrostatic channel champions a new understanding of the ecology of arthropods and reveals previously unknown ecological relationships and novel interactions between species.

### 8.2. Future work

To date, the primary candidate for electrosensors is mechanosensory filifrom hairs [7,9], yet we hasten to add that other forms of electrosensors are likely to exist. Furthermore, there is a growing body of experimental evidence demonstrating the behavioural implications of electroreception and the detection of weak electrical fields. This evidence includes work on spiders [5], bumblebees [3,42], honeybees [4,43], ticks [44] and hoverflies [6].

Given the above, further empirical evidence of the electrical sensitivity and mechanics of sensory hairs (and indeed other sensors) is required to assess the relevance of the theory presented here, and the utility of electroreception in general. Within our laboratory, we are conducting such experiments, measuring and characterizing hair deflections in several arthropod species. The publication of these results is forthcoming, including integration between the theoretical and empirical work. For further comments on the directions and requirements for further empirical evidence, see [21]. Nonetheless, the presented work shows unique sensory possibilities that electroreception affords and indicates promising directions in which to pursue empirical research.

Overall, several avenues for future work emerge from this investigation. For example, modelling larger arrays of hairs and biologically relevant substrates and scenarios will uncover further nuances of bimodality in sensory hairs. Furthermore, a broader investigation of aerodynamic effects is required. Alongside the electrostatic coupling, closely located hairs influence each other through the local fluid medium, known as viscous-mediated coupling [16,18,45]. The comparative strengths of these forms of coupling need to be theoretically and experimentally examined for a host of different situations. These include scenarios of aerodynamic wingbeat signals and impulses from a moving arthropod.

## Data Availability

Please use the following link to access the data used for figure 1: https://doi.org/10.5061/dryad.80gb5mkw8. The data are provided in the electronic supplementary material [46].

## References

[1] Crampton WG. 2019 Electroreception, electrogenesis and electric signal evolution. J. Fish Biol. **95**, 92-134. (10.1111/jfb.2019.95.issue-1) 30729523

[2] Steullet P, Edwards DH, Derby CD. 2007 An electric sense in crayfish? Biol. Bull. **213**, 16-20. (10.2307/25066614) 17679716

[3] Clarke D, Whitney H, Sutton G, Robert D. 2013 Detection and learning of floral electric fields by bumblebees. Science **340**, 66-69. (10.1126/science.1230883) 23429701

[4] Greggers U, Koch G, Schmidt V, Dürr A, Floriou-Servou A, Piepenbrock D, Göpfert MC, Menzel R. 2013 Reception and learning of electric fields in bees. Proc. R. Soc. B **280**, 20130528. (10.1098/rspb.2013.0528) PMC3619523 23536603

[5] Morley EL, Robert D. 2018 Electric fields elicit ballooning in spiders. Curr. Biol. **28**, 2324-2330.e2. (10.1016/j.cub.2018.05.057) 29983315 PMC6065530

[6] Khan SA et al. 2021 Electric field detection as floral cue in hoverfly pollination. Sci. Rep. **11**, 1-9. (10.1038/s41598-020-79139-8) 34548579 PMC8455601

[7] Sutton GP, Clarke D, Morley EL, Robert D. 2016 Mechanosensory hairs in bumblebees (*Bombus terrestris*) detect weak electric fields. Proc. Natl Acad. Sci. USA **113**, 7261-7265. (10.1073/pnas.1601624113) 27247399 PMC4932954

[8] Hunting ER, O’Reilly LJ, Harrison RG, Manser K, England SJ, Harris BH, Robert D. 2022 Observed electric charge of insect swarms and their contribution to atmospheric electricity. Iscience **25**, 105241. (10.1016/j.isci.2022.105241) 36439985 PMC9684032

[9] England SJ, Robert D. 2021 The ecology of electricity and electroreception. Biol. Rev. **97**, 383-413. (10.1111/brv.v97.1) 34643022

[10] Koh K, Robert D. 2020 Bumblebee hairs as electric and air motion sensors: theoretical analysis of an isolated hair. J. R. Soc. Interface **17**, 20200146. (10.1098/rsif.2020.0146) 32634368 PMC7423416

[11] Palmer RA, Chenchiah IV, Robert D. 2021 Analysis of aerodynamic and electrostatic sensing in mechanoreceptor arthropod hairs. J. Theor. Biol. **530**, 110871. (10.1016/j.jtbi.2021.110871) 34411607

[12] Palmer RA, Chenchiah IV, Robert D. 2022 The mechanics and interactions of electrically sensitive mechanoreceptive hair arrays of arthropods. J. R. Soc. Interface **19**, 20220053. (10.1098/rsif.2022.0053) 35317646 PMC8941402

[13] Casas J, Dangles O. 2010 Physical ecology of fluid flow sensing in arthropods. Annu. Rev. Entomol. **55**, 505-520. (10.1146/ento.2010.55.issue-1) 19743914

[14] Tautz J, Markl H. 1978 Caterpillars detect flying wasps by hairs sensitive to airborne vibration. Behav. Ecol. Sociobiol. **4**, 101-110. (10.1007/BF00302564)

[15] Shimozawa T, Kumagai T, Baba Y. 1998 Structural scaling and functional design of the cercal wind-receptor hairs of cricket. J. Comp. Physiol. A **183**, 171-186. (10.1007/s003590050245)

[16] Bathellier B, Barth FG, Albert JT, Humphrey JA. 2005 Viscosity-mediated motion coupling between pairs of trichobothria on the leg of the spider *Cupiennius salei*. J. Comp. Physiol. A **191**, 733-746. (10.1007/s00359-005-0629-5) 16041533

[17] Humphrey JA, Barth FG. 2007 Medium flow-sensing hairs: biomechanics and models. Adv. Insect Physiol. **34**, 1-80. (10.1016/S0065-2806(07)34001-0)

[18] Cummins B, Gedeon T, Klapper I, Cortez R. 2007 Interaction between arthropod filiform hairs in a fluid environment. J. Theor. Biol. **247**, 266-280. (10.1016/j.jtbi.2007.02.003) 17434184 PMC2742163

[19] Barth FG, Höller A. 1999 Dynamics of arthropod filiform hairs. V. The response of spider trichobothria to natural stimuli. Phil. Trans. R. Soc. B **354**, 183-192. (10.1098/rstb.1999.0370)

[20] Steinmann T, Casas J. 2017 The morphological heterogeneity of cricket flow-sensing hairs conveys the complex flow signature of predator attacks. J. R. Soc. Interface **14**, 20170324. (10.1098/rsif.2017.0324) 28637919 PMC5493808

[21] Palmer RA, Chenchiah IV, Robert D. 2023 Passive electrolocation in terrestrial arthropods: theoretical modelling of location detection. J. Theor. Biol. **558**, 111357. (10.1016/j.jtbi.2022.111357) 36410450 PMC10338892

[22] Clarke D, Morley E, Robert D. 2017 The bee, the flower, and the electric field: electric ecology and aerial electroreception. J. Comp. Physiol. A **203**, 737-748. (10.1007/s00359-017-1176-6) PMC5599473 28647753

[23] Montgomery C, Koh K, Robert D, 2019 Measurement of electric charges on foraging bumblebees (*Bombus terrestris*). J. Phys. Conf. Ser. **1322**, 012002. IOP Publishing. (10.1088/1742-6596/1322/1/012002)

[24] Kumagai T, Shimozawa T, Baba Y. 1998 The shape of wind-receptor hairs of cricket and cockroach. J. Comp. Physiol. A **183**, 187-192. (10.1007/s003590050246)

[25] Khan KA, Liu T. 2022 Morphological structure and distribution of hairiness on different body parts of *Apis mellifera* with an implication on pollination biology and a novel method to measure the hair length. Insects **13**, 189. (10.3390/insects13020189) 35206762 PMC8874558

[26] Koh K, Robert D. 2020 Bumblebee hairs as electric and air motion sensors: theoretical analysis of an isolated hair. J. R. Soc. Interface **17**, 20200146. (10.1098/rsif.2020.0146) 32634368 PMC7423416

[27] Bathellier B, Steinmann T, Barth FG, Casas J. 2012 Air motion sensing hairs of arthropods detect high frequencies at near-maximal mechanical efficiency. J. R. Soc. Interface **9**, 1131-1143. (10.1098/rsif.2011.0690) 22171067 PMC3350735

[28] Shimozawa T, Murakami J, Kumagai T. 2003 Cricket wind receptors: thermal noise for the highest sensitivity known. In *Sensors and sensing in biology and engineering*, pp. 145–157. Berlin, Germany: Springer.

[29] Barth FG, Wastl U, Humphrey JA, Devarakonda R. 1993 Dynamics of arthropod filiform hairs. II. Mechanical properties of spider trichobothria (*Cupiennius salei* Keys). Phil. Trans. R. Soc. B **340**, 445-461. (10.1098/rstb.1993.0084)

[30] Humphrey JA, Barth FG. 2007 Medium flow-sensing hairs: biomechanics and models. Adv. Insect Physiol. **34**, 1-80. (10.1016/S0065-2806(07)34001-0)

[31] Cummins B, Gedeon T, Klapper I, Cortez R. 2007 Interaction between arthropod filiform hairs in a fluid environment. J. Theor. Biol. **247**, 266-280. (10.1016/j.jtbi.2007.02.003) 17434184 PMC2742163

[32] Steinmann T, Casas J. 2017 The morphological heterogeneity of cricket flow-sensing hairs conveys the complex flow signature of predator attacks. J. R. Soc. Interface **14**, 20170324. (10.1098/rsif.2017.0324) 28637919 PMC5493808

[33] Kumagai T, Shimozawa T, Baba Y. 1998 The shape of wind-receptor hairs of cricket and cockroach. J. Comp. Physiol. A **183**, 187-192. (10.1007/s003590050246)

[34] Robert D, Amoroso J, Hoy RR. 1992 The evolutionary convergence of hearing in a parasitoid fly and its cricket host. Science **258**, 1135-1137. (10.1126/science.1439820) 1439820

[35] Holderied M, Korine C, Moritz T. 2011 Hemprich’s long-eared bat (*Otonycteris hemprichii*) as a predator of scorpions: whispering echolocation, passive gleaning and prey selection. J. Comp. Physiol. A **197**, 425-433. (10.1007/s00359-010-0608-3) 21086132

[36] Dean T. 2003 *Fastest Flyer*. In: *Book of Insect Records*. Gainsville, FL: The University of Florida.

[37] Roquer-Beni L, Rodrigo A, Arnan X, Klein A-M, Fornoff F, Boreux V, Bosch J. 2020 A novel method to measure hairiness in bees and other insect pollinators. Ecol. Evol. **10**, 2979-2990. (10.1002/ece3.v10.6) 32211170 PMC7083657

[38] Magal C, Dangles O, Caparroy P, Casas J. 2006 Hair canopy of cricket sensory system tuned to predator signals. J. Theor. Biol. **241**, 459-466. (10.1016/j.jtbi.2005.12.009) 16427653

[39] Reber T, Baird E, Dacke M. 2016 The final moments of landing in bumblebees, *Bombus terrestris*. J. Comp. Physiol. A **202**, 277-285. (10.1007/s00359-016-1073-4) 26868924

[40] Humphrey JA, Devarakonda R, Iglesias I, Barth FG. 1993 Dynamics of arthropod filiform hairs. I. Mathematical modelling of the hair and air motions. Phil. Trans. R. Soc. B **340**, 423-444. (10.1098/rstb.1993.0083)

[41] Palmer RA, Chenchiah IV, Robert D. 2021 Analysis of aerodynamic and electrostatic sensing in mechanoreceptor arthropod hairs. J. Theor. Biol. **530**, 110871. (10.1016/j.jtbi.2021.110871) 34411607

[42] Hunting ER, England SJ, Koh K, Lawson DA, Brun NR, Robert D. 2022 Synthetic fertilizers alter floral biophysical cues and bumblebee foraging behavior. PNAS Nexus **1**, pgac230. (10.1093/pnasnexus/pgac230) 36712354 PMC9802097

[43] Amador GJ, Matherne M, Waller D, Mathews M, Gorb SN, Hu DL. 2017 Honey bee hairs and pollenkitt are essential for pollen capture and removal. Bioinspir. Biomim. **12**, 026015. (10.1088/1748-3190/aa5c6e) 28332480

[44] England SJ, Lihou K, Robert D. 2023 Static electricity passively attracts ticks onto hosts. Curr. Biol. **33**, 3041–3047. 10.1016/j.cub.2023.06.021 PMC7616434 37392744

[45] Krijnen GJ, Steinmann T, Jaganatharaja RK, Casas J. 2019 Insect-inspired distributed flow-sensing: fluid-mediated coupling between sensors. In *Architectured materials in nature and engineering*, pp. 355–392. Berlin, Germany: Springer.

[46] Palmer RA, O’Reilly LJ, Carpenter J, Chenchiah IV, Robert D. 2023 An analysis of time-varying dynamics in electrically-sensitive arthropod hairs to understand real-world electrical sensing. Figshare. (10.6084/m9.figshare.c.6753791)

